# Voltage-sensing phosphatase modulation by a C2 domain

**DOI:** 10.3389/fphar.2015.00063

**Published:** 2015-04-08

**Authors:** Paul M. Castle, Kevin D. Zolman, Susy C. Kohout

**Affiliations:** Department of Cell Biology and Neuroscience, Montana State UniversityBozeman, MT, USA

**Keywords:** voltage-sensing phosphatase, C2 domain, PIP, PH domains, voltage clamp fluorometry, membrane potential

## Abstract

The voltage-sensing phosphatase (VSP) is the first example of an enzyme controlled by changes in membrane potential. VSP has four distinct regions: the transmembrane voltage-sensing domain (VSD), the inter-domain linker, the cytosolic catalytic domain, and the C2 domain. The VSD transmits the changes in membrane potential through the inter-domain linker activating the catalytic domain which then dephosphorylates phosphatidylinositol phosphate (PIP) lipids. The role of the C2, however, has not been established. In this study, we explore two possible roles for the C2: catalysis and membrane-binding. The Ci-VSP crystal structures show that the C2 residue Y522 lines the active site suggesting a contribution to catalysis. When we mutated Y522 to phenylalanine, we found a shift in the voltage dependence of activity. This suggests hydrogen bonding as a mechanism of action. Going one step further, when we deleted the entire C2 domain, we found voltage-dependent enzyme activity was no longer detectable. This result clearly indicates the entire C2 is necessary for catalysis as well as for modulating activity. As C2s are known membrane-binding domains, we tested whether the VSP C2 interacts with the membrane. We probed a cluster of four positively charged residues lining the top of the C2 and suggested by previous studies to interact with phosphatidylinositol 4,5-bisphosphate [PI(4,5)P_2_] (Kalli et al., 2014). Neutralizing those positive charges significantly shifted the voltage dependence of activity to higher voltages. We tested membrane binding by depleting PI(4,5)P_2_ from the membrane using the 5HT2C receptor and found that the VSD motions as measured by voltage clamp fluorometry (VCF) were not changed. These results suggest that if the C2 domain interacts with the membrane to influence VSP function it may not occur exclusively through PI(4,5)P_2_. Together, this data advances our understanding of the VSP C2 by demonstrating a necessary and critical role for the C2 domain in VSP function.

## Introduction

**Figure 1:**
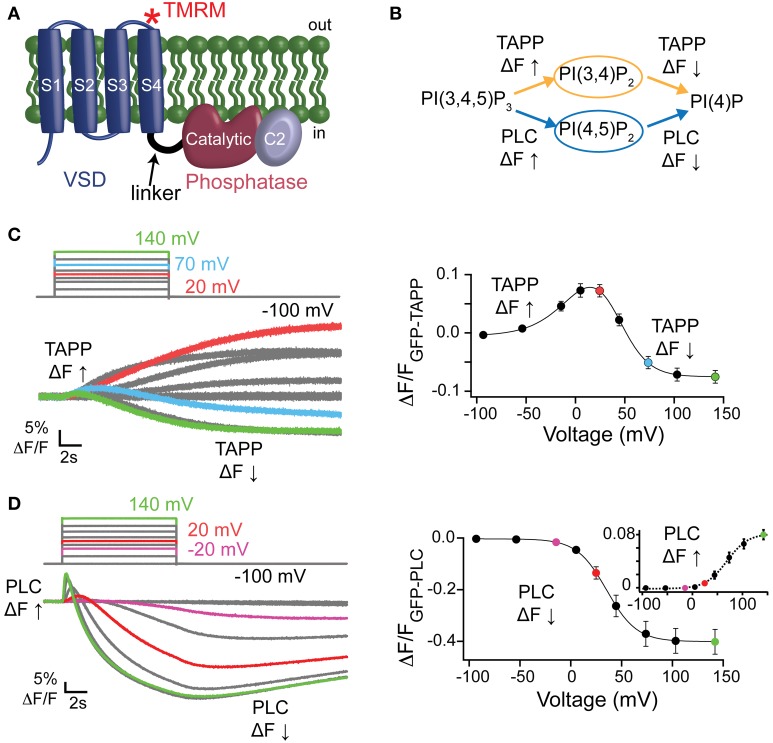
**Activity of Ci-VSP in oocytes using PIP sensitive PH domains**. **(A)** Cartoon of VSP with the VSD, linker, catalytic and C2 domains labeled. Asterisk depicts location of the TMRM probe (G214C-TMRM). **(B)** VSP reactions where the PI(3,4)P_2_ sensitive GFP-TAPP-PH monitors the upper reactions (orange) and the PI(4,5)P_2_ sensitive GFP-PLC-PH monitors the lower reactions (blue). (**C**, left) Representative GFP-TAPP-PH traces for WT^*^ Ci-VSP during several voltage steps from a holding potential (hp) of −100 mV and with recovery time between each step. A fluorescence increase indicates an increase of PI(3,4)P_2_ on the membrane while a fluorescence decrease indicates a decrease of PI(3,4)P_2_ on the membrane; 20 mV = red, 70 mV = cyan, 140 mV = green. The fluorescence may keep changing even after the end of the voltage step. This is a function of the affinity of the PH domain and not an indication of voltage independent Ci-VSP activity. (**C**, right) ΔF/F TAPP fluorescence vs. voltage relationship (FV) with the fluorescence increase (net 5-phosphatase reaction) predominating at lower voltages and the fluorescence decrease (net 3-phosphatase reaction) predominating at higher voltages. Data fit with a double Boltzmann equation. (**D**, left) Representative traces of GFP-PLC-PH co-expressed with WT^*^ during several voltage steps as in **(C)**. The fluorescence increase indicates an increase of PI(4,5)P_2_ on the membrane while the fluorescence decrease indicates a decrease of PI(4,5)P_2_ on the membrane; −20 mV = purple, 20 mV = red, 140 mV = green. (**D**, right) ΔF/F PLC FV with the fluorescence decrease (FV Down, net 5-phosphatase reaction, solid line) and the fluorescence increase (FV Up, 3-phosphatase reaction, dashed line, inset) separated. Data fit with a Boltzmann equation. All error bars are ± s.e.m; n ≥ 16. Some errors bars are smaller than the size of the symbols.

The voltage-sensing phosphatase (VSP) is the first enzyme controlled by the membrane potential of the cell (Murata et al., 2005). Like all voltage-regulated proteins, VSP has a voltage-sensing domain (VSD) with four transmembrane helices and sensing charges on the fourth helix, S4. The phosphatase domain of VSP is homologous to the “phosphatase and tensin homolog deleted on chromosome 10” (PTEN) protein, a lipid and protein phosphatase (Murata et al., 2005; Okamura and Dixon, 2011; Villalba-Galea, 2012). The VSP phosphatase domain can be separated into three regions of interest; the inter-domain linker, the catalytic domain, and the C2 domain (Figure 1A). The linker has been implicated in coupling the VSD to the phosphatase domain in a lipid-dependent manner (Murata et al., 2005; Villalba-Galea et al., 2009; Kohout et al., 2010; Hobiger et al., 2012, 2013; Liu et al., 2012). The VSP catalytic domain is 44% identical with that of PTEN which fueled the assumption that VSP and PTEN catalyze the same reaction, dephosphorylating phosphatidylinositol 3,4,5-trisphosphate [PI(3,4,5)P_3_] at the 3-position (Murata et al., 2005). Subsequent studies have found VSP dephosphorylates three different phosphatidylinositol phosphate (PIP) substrates at two positions: PI(3,4,5)P_3_ and PI(4,5)P_2_ at the 5-phosphate as well as phosphatidylinositol 3,4-bisphosphate [PI(3,4)P_2_] at the 3-phosphate, all in a voltage-dependent manner (Figure 1B) (Iwasaki et al., 2008; Halaszovich et al., 2009; Kurokawa et al., 2012). The potential dephosphorylation of PI(3,4,5)P_3_ at the 3-phosphate by VSP remains unsettled. Even so, VSP clearly has a broader activity range than PTEN, and as a result, could have an even broader impact on PIP regulation.

PIP regulation is critical to a large number of cellular processes including cell proliferation, migration, development, synaptic regulation, ion channel modulation, and others (Di Paolo and De Camilli, 2006; Logothetis et al., 2010; Koch and Holt, 2012; Balla, 2013). PIP concentrations are tightly controlled and a small deviation initiates the up- or down-regulation of the appropriate kinase or phosphatase to take the concentrations back to normal levels. Several human diseases are linked to mutations in PIP-metabolizing enzymes, including cancers, peripheral neuropathy, Alzheimer's, and psychiatric disorders such as bipolar disorder (Simpson and Parsons, 2001; Di Paolo and De Camilli, 2006; McCrea and De Camilli, 2009; Aguissa-Touré and Li, 2012). Because of the role different PIPs play in both normal cellular regulation and in disease states, it is crucial to understand how all PIPs are interconverted. The role of VSP in this process is particularly important since it serves as both a 5- and a 3-phosphatase.

Much of the VSP research focus has been on the VSD, linker and the catalytic domains, leaving the contribution of the VSP C2 domain unclear (Figure 1A). Traditionally, C2 domains are independently folded, lipid-binding domains (Lemmon, 2008). While C2s are not well-conserved on the sequence level, they are very well-conserved on the structural level, having a characteristic eight-stranded anti-parallel β-sandwich with three loops on one end responsible for binding calcium [Ca^2+^-binding loops (CBLs) 1–3] (Nalefski and Falke, 1996; Lemmon, 2008). The VSP C2 does not have the required aspartic acids for binding Ca^2+^ so it is unlikely to be regulated by Ca^2+^, much like the Ca^2+^ independent C2 of PTEN. Not all C2s bind Ca^2+^ yet many still play a role in signaling, including membrane-binding (Corbalán-García et al., 2003; Sánchez-Bautista et al., 2007) and mediating protein–protein interactions (Johnson et al., 1996; Schechtman et al., 2004) as well as regulating ion channels (Miki et al., 2007). Hence even without the ability to bind Ca^2+^, C2s are actively involved in signaling.

The VSP high-resolution crystal structures suggest a new potential role for C2 domains—contributing to catalysis. A tyrosine (Tyr) from the C2 (Y522) was found physically in the active site of the catalytic domain (Matsuda et al., 2011; Liu et al., 2012). Supporting that hypothesis, the mutation Y522A altered catalysis by slowing down the rate of 3-phosphate dephosphorylation of PI(3,4)P_2_ (Liu et al., 2012). The recent cloning of salamander VSP also suggests the VSP C2 domains are important since it is missing part of the C2 and is catalytically dead (Mutua et al., 2014). However, very little is known regarding the contributions and mechanisms of the VSP C2.

To fill that gap, we investigated how the VSP C2 domain contributes to function by deleting the entire C2 domain as well as mutating key residues. We first evaluated wild type Ci-VSP by using pleckstrin homology (PH) domains fused to green fluorescent protein (GFP) and testing multiple voltage steps to map the voltage dependence of each possible Ci-VSP reaction (Figure 1B). We found evidence supporting the dephosphorylation of PI(3,4,5)P_3_ at the 3-phosphate indicating that Ci-VSP catalyzes a total of four reactions using three different substrates. With that new baseline of activity, we probed the contribution of Y522 and found that both the hydrogen bonding and the size of Tyr modulate activity. We found the full C2 domain is necessary for function because the C2 deletion diminished catalytic activity to control levels. We also found that neutralizing a cluster of positive charges on the C2 domain dramatically shifted the voltage dependence of activity suggesting an interaction with negatively charged lipids. Together our results support a dual function of the C2 domain in catalysis and membrane-binding.

## Materials and methods

### Molecular biology

The Ci-VSP in the pSD64TF vector was kindly provided by Y. Okamura (Osaka University). GFP-PLC-PH was kindly provided by T. Meyer (Stanford University) and subcloned into pGEMHE vectors. GFP-TAPP-PH was kindly provided by T. Balla (NIH) and subcloned into pGEMHE. All mutants were made using PfuTurbo polymerase (Agilent). All DNA was confirmed by DNA sequencing. RNA was transcribed using T7 and SP6 mMessage mMachine (Ambion) kits.

### Voltage clamp fluorometry

Voltage clamp fluorometry (VCF) was performed as described previously (Kohout et al., 2010). Briefly, surgically removed *Xenopus laevis* oocytes were injected with 50 nl mRNA at 0.06–1.8 μg μl^−1^ depending on the experiment. Cells were then incubated in ND-96 (96 mM NaCl, 2 mM KCl, 1.8 mM CaCl_2_, 1 mM MgCl_2_, 50 mg ml^−1^ gentamicin, 2.5 mM Na pyruvate, and 5 mM HEPES, pH 7.6) at 18°C for 1–4 days.

A Leica DM IRBE inverted microscope with a Leica HC Pl APO 20×/0.7 fluorescence objective was used with a Dagan CA-1B amplifier (Dagan Corporation) and illuminated with a Lumen Dynamics X-Cite XLED1 light source. Intensity was measured with a ThorLabs photomultiplier tube. The amplifier and LED were controlled by a Digidata-1440A board and pClamp10.3 software package (Axon Instruments). For TMRM experiments, light was filtered through an HQ531/40 excitation filter, an HQ593/40 emission filter and a Q562LP dichroic (Semrock). Fluorescence signals were low-pass filtered at 2 kHz through an eight-pole Bessel filter (Frequency Devices).

On the day of the experiment, cells were incubated in a high-potassium solution (92 mM KCl, 0.75 mM CaCl_2_, 1 mM MgCl_2_, 10 mM HEPES, pH7.5) with 25 μM tetramethylrhodamine-6-maleimide (Invitrogen) for 30 min on ice and in the dark. After extensive washing with ND-96, the cells were stored in ND-96′ (ND-96 without gentamicin or pyruvate), in the dark and at 10–19°C until the time of the experiment. ND-96′ was used for the recording solution. In all experiments, only cells with good control of voltage were analyzed. All reported voltages and voltage steps are actual measurements. For VCF experiments, the voltage protocol consisted of 10 mV voltage steps starting at −150 mV and ending at 200 mV. The resulting fluorescence was then plotted vs. the voltage to generate the fluorescence/voltage relationship (FV).

For experiments with the 5HT2C receptor, an RNA ratio of 40:1 was used for experiments co-expressing Ci-VSP and the receptor (total RNA ~0.82 μg μl^−1^). An initial FV protocol (*t* = 0, voltage steps from −150 to 200 mV in 10 mV increments) was followed by perfusion of 10 μM serotonin (Sigma) for 5–10 s while recording the Ca^2+^-activated chloride channels. The cell was then perfused with ND-96′ until the final FV protocol at *t* = 10 min.

### Fluorescent measurements of activity

RNA ratios ranging from 2:1 to 60:1 μg μl^−1^ were used for Ci-VSP and each PH domain (total RNA 0.08–1.43 μg μl^−1^). Cells were recorded in perfused ND-96′. GFP-PLC-PH and GFP-TAPP-PH were used to detect PI(4,5)P_2_ and PI(3,4)P_2_, respectively, using a GFP filter set (Chroma) consisting of a 470/40 excitation filter, a 525/50 emission filter, and a 495 dichroic. For the TAPP-PH, 8 μM insulin was added to the ND-96′ to promote PI3K activity and up regulate PI(3,4,5)P_3_ levels. Ci-VSP expression levels were confirmed in each oocyte by TMRM labeling and measurement of the TMRM fluorescence change induced by VSD motion. Other conditions were the same as for the VCF. For PH domain FV experiments, the protocol consisted of voltage steps from −100 mV to +140 or +180 mV in irregular increments. Rest periods of 1.5–5 min between voltage steps were used to allow the cell to recover depleted PIP concentrations before starting the next voltage step. The resulting fluorescence was then plotted vs. the voltage to generate the FV relationship.

### Data analysis

Kinetic and steady state fluorescence traces were analyzed using Clampfit (Molecular Devices), IGOR Pro (WaveMetrics), and Excel (Microsoft). Steady state voltage-dependent traces were fit with either single or double Boltzmann equations. TMRM data was normalized to the maximum amplitude of the Boltzmann fits, while the PH domain data is shown as ΔF/F. Error bars indicate the standard error of the mean (s.e.m.). Statistical significance was determined using the Student's *t*-test.

## Results

### Wild type activity

To determine VSP activity, we used PH domains that bind specific PIPs and that are linked to GFP, allowing us to monitor PIP concentrations by tracking fluorescence on the membrane. This PH domain assay has been used before to test for Ci-VSP activity (Halaszovich et al., 2009; Sakata et al., 2011; Kurokawa et al., 2012; Liu et al., 2012) and has the advantage of being able to separate out the different possible dephosphorylation reactions catalyzed by Ci-VSP, unlike channels such as IRK or KCNQ that are known to only be dependent on PI(4,5)P_2_ and thus only test for PI(4,5)P_2_ concentrations. Using *Xenopus laevis* oocytes, we expressed two different GFP-PH domains with Ci-VSP, the tandem PH domain-containing protein 1 PH domain (GFP-TAPP-PH) and the phospholipase C PH domain (GFP-PLC-PH). These two PH domains allow us to monitor both 5- and 3-phosphatase reactions attributed to Ci-VSP because TAPP-PH binds to PI(3,4)P_2_ (Kimber et al., 2002; Manna et al., 2007) while PLC-PH binds to PI(4,5)P_2_ (Stauffer et al., 1998; Várnai and Balla, 1998) (Figure 1B). When the 5-phosphate is removed from PI(3,4,5)P_3_ leaving PI(3,4)P_2_ as the product, GFP-TAPP-PH will go to the membrane, increasing the membrane fluorescence, ΔF↑ (Figure 1B). When the 3-phosphate from PI(3,4)P_2_ is removed the fluorescence will decrease as GFP-TAPP-PH comes off the membrane, ΔF↓ (Figure 1B). GFP-PLC-PH monitors the second set of reactions by going to the membrane when the 3-phosphate is removed from PI(3,4,5)P_3_ leaving PI(4,5)P_2_ as the product, causing the fluorescence to increase, ΔF↑ (Figure 1B). GFP-PLC-PH will come off the membrane when the 5-phosphate is removed from PI(4,5)P_2_ leaving PI(4)P, causing a fluorescence decrease, ΔF↓ (Figure 1B). Both reaction pathways end with PI(4)P, however distinguishing the two is important because all three substrates, PI(3,4,5)P_3_, PI(3,4)P_2_, and PI(4,5)P_2_, are known to regulate different functions in the cell (Balla, 2013).

It is important to note that the reactions observed using the PH domains are all net reactions. For example, an increase of GFP-TAPP-PH to the membrane indicates a net increase of PI(3,4)P_2_ at the membrane. Other processes within the cell, including the kinases responsible for phosphorylating PIPs, will influence the relative concentrations of PIPs during the assay. These processes obscure the actual kinetics of the reactions due to Ci-VSP activity. However, no other process influencing the PIP concentrations in the cell is directly voltage dependent and the overall concentration of PI(3,4)P_2_ must increase for GFP-TAPP-PH to accumulate on the membrane. Therefore, the simplest interpretation of a voltage-dependent increase of GFP-TAPP-PH is an indication of Ci-VSP voltage-dependent 5-phoshpatase activity. At higher voltages, the fluorescence decrease of GFP-TAPP-PH is likewise a mixture of reactions. Because the decrease dominates the signal, the simplest interpretation is an increase in the 3-phosphatase activity of VSP. The same is true for the GFP-PLC-PH domain assay.

First, we tested the PI(3,4)P_2_ pathway by co-expressing GFP-TAPP-PH with Ci-VSP in *X. laevis* oocytes. To confirm the expression of Ci-VSP, all proteins presented here have the G214C mutation in the VSD allowing us to label the VSD with a fluorophore, tetramethylrhodamine maleimide (TMRM) (Figure 1A). Thus, wild type Ci-VSP (WT^*^) is G214C Ci-VSP labeled with TMRM. The TMRM fluorescence was used to establish Ci-VSP expression before testing activity using VCF (data not shown). To map out the voltage dependence of each possible dephosphorylation reaction, we first tested WT^*^ with a wide range of voltages, from −100 to +140 mV using two-electrode voltage clamp. At lower voltages, we observed an increase of GFP-TAPP-PH to the membrane suggesting the expected 5-phosphatase activity of WT^*^ (Figure 1C, left, red). We used short voltage steps of 10 s to allow for complete recovery of the PIP concentrations between each voltage step. As a result of the short steps, the fluorescence may continue to change after the voltage step has turned off. This is a function of the affinity of the PH domain and not an indication that VSP is still active. At intermediate voltages, both an increase and a decrease in fluorescence are visible indicating that both 5- and 3-phosphatase activities are observable (Figure 1C, left, cyan). At higher voltages, a fluorescence decrease predominates suggesting the 3-phosphatase activity of WT^*^ (Figure 1C, left, green).

By plotting the fluorescence at the end of each voltage step vs. the voltage of each step (TAPP FV), we show a clear voltage dependence of each reaction (Figure 1C, right). We observed increasing fluorescence from −100 mV until 20 mV (called here TAPP FV Up), indicating more PI(3,4)P_2_ at the membrane suggesting Ci-VSP 5-phosphatase activity. At 70 mV and above, the majority of the signal is a decrease in fluorescence (called here TAPP FV Down), indicating less PI(3,4)P_2_ at the membrane suggesting Ci-VSP 3-phosphatase activity (Figure 1C, right). Between 20 and 70 mV, we observe a mixture of increasing and decreasing fluorescence within the same voltage step indicating that both 5- and 3-phosphatase reactions can occur at the same voltage. Using a double Boltzmann equation to fit the FV from each cell, we were able to separate out the *V*_1/2_ for the fluorescence increase from the *V*_1/2_ for the fluorescence decrease (Table 1). Note, the reactions behind the changes in fluorescence are in equilibrium with each other thus we are measuring the net PI(3,4)P_2_ concentration at each voltage. We cannot rule out simultaneous 5- and 3-phosphatase VSP activity at the lower or higher voltage. We are limited to interpreting the net fluorescence change where a decrease suggests the production of PI(3,4)P_2_ and an increase suggests the dephosphorylation of PI(3,4)P_2_. Our double Boltzmann fits indicate a *V*_1/2_ for the fluorescence decrease of 44 ± 3, which is higher than for the fluorescence increase, 9 ± 5, suggesting that the 3-phosphatase predominates at higher voltages while the 5-phosphatase reaction predominates at the lower voltages. Our results agree with previous results indicating that voltage may control the substrate specificity of Ci-VSP (Kurokawa et al., 2012).

**Table 1:** **Voltage dependence of activity**.

**VSP**	**TAPP FV fits**			**PLC FV fits**		
	***n***	***V*_1/2_ up**	***V*_1/2_ down**	***n***	***V*_1/2_ down**	***V*_1/2_ up**
WT^*^	24	9 ± 5	44 ± 3	16	37 ± 4	70 ± 5
Y522A^*^	8	18 ± 6	51 ± 4	7	47 ± 4	87 ± 6^*^
Y522F^*^	8	27 ± 5^*^	58 ± 4^**^	6	58 ± 5^*^	94 ± 6^**^
CS^*^	18	60 ± 1	n/a	16	n/a^@^	n/a
ΔC2^*^	9	64 ± 5^#^	n/a	9	n/a^@^	n/a
QuadK^*^	6	50 ± 9^*^	99 ± 5^***^	7	68 ± 4^***^	115 ± 5^***^
K553Q^*^	6	32 ± 2^***^	53 ± 2^*^	6	49 ± 7	79 ± 7
K554Q^*^	6	14 ± 6	52 ± 8	7	37 ± 4	63 ± 3
K555Q^*^	6	38 ± 9^*^	72 ± 8^*^	6	58 ± 12	89 ± 13
K558Q^*^	6	15 ± 3	39 ± 3	6	40 ± 6	77 ± 5

Average V_1/2_ from double Boltzmann fits to each cell (n) for the TAPP FVs and single Boltzmann fits for the PLC FVs. Student t-tests,

* p ≤ 0.05;

** p ≤ 0.01;

*** p ≤ 0.001 (compared to WT^*^).

# Not significant (compared to CS^*^); n/a, no data to fit; n/a

@ *, amplitude too small to fit*.

Switching to the PI(4,5)P_2_ pathway, we co-expressed WT^*^ with GFP-PLC-PH and observed a dominant decrease in fluorescence starting at lower voltages (Figure 1D, left, purple) indicating 5-phosphatase activity. A short fluorescence increase clearly develops at higher voltages (Figure 1D, left, red and green) though the overall change is still a decrease. This fluorescence increase suggests PLC is also following two reactions where the fluorescence increase indicates more PI(4,5)P_2_ on the membrane while the fluorescence decrease indicates less PI(4,5)P_2_ on the membrane. The fluorescence decrease dominates at all voltages obscuring the shorter-lived fluorescence increase. To separate the 5- vs. 3-phosphatase reactions, we plotted the GFP-PLC-PH fluorescence decrease vs. voltage relationship (PLC FV Down, Figure 1D, right) separately from the fluorescence increase vs. voltage relationship (PLC FV Up, Figure 1D, right, inset). We fit the FVs from individual cells using single Boltzmann equations to determine the voltage dependence of each reaction. Using these PLC FVs, we found that the 5-phosphatase activity started at −20 mV with a *V*_1/2_ of 37 ± 4 while the 3-phosphatase activity was not visible until ≥ 20 mV with a *V*_1/2_ of 70 ± 5 (Figure 1D, right, Table 1), suggesting Ci-VSP is capable of dephosphorylating PI(3,4,5)P_3_ at the 3-phosphate, but it only appears to dominate the reactions under higher voltage conditions. The apparent dominance of the 3-phosphatase reaction at higher voltages for two independent reporters suggests that the voltage may control the specificity of the substrate in agreement with previous studies (Kurokawa et al., 2012). We next turned to testing the contributions of the C2 domain by comparing C2 mutations to wild type.

### C2 domain contribution to activity via the third inactivated calcium-binding loop

**Figure 2:**
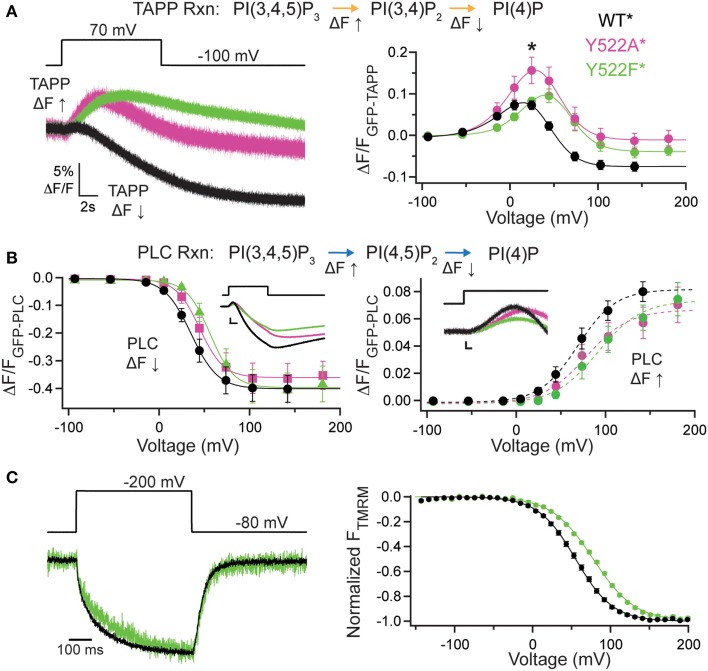
**Third inactivated calcium-binding loop from C2 domain shifts voltage dependence of VSP activity**. (**A**, left) Representative GFP-TAPP-PH trace during a step from an hp of −100 mV to +70 mV for WT^*^ (black), Y522A^*^ (magenta) and Y522F^*^ (green). Both Y522A^*^ and Y522F^*^ show a similar fluorescence increase and decrease during the course of the voltage step indicating both PI(3,4)P_2_ production and depletion while WT^*^ shows mainly a fluorescence decrease at this voltage, indicating PI(3,4)P_2_ depletion. TAPP-PH reaction listed on top. (**A**, right) ΔF/F TAPP FV. Both Y522A^*^ and Y522F^*^ show pronounced fluorescence decreases at higher voltages, indicating both cause PI(3,4)P_2_ depletion at higher voltages. Y522F^*^ shifted the voltage dependence of activity by 14–18 mV (Table 1) and Y522A^*^ increased the peak amplitude of the response (Student *t*-test, ^*^p ≤ 0.05), suggesting the importance of both the size and hydrogen bonding of position 522, *n* ≥ 8. Data fit with a double Boltzmann. (**B**, left) ΔF/F PLC FV Down. The fluorescence decrease FV (net 5-phosphatase activity) from GFP-PLC-PH fit with a Boltzmann. Inset shows 40 mV step representative traces. Scale bars for inset are 5% ΔF/F and 2 s. Colors as in **(A)**. Y522F^*^ shifted the voltage dependence by 21 mV (Table 1). PLC-PH reaction listed on top. (**B**, right) ΔF/F PLC FV Up. The fluorescence increase FV (net 3-phosphatase activity) from the same GFP-PLC-PH data as in (**B**, left), fit with a Boltzmann. Inset shows enlarged 40 mV step representative traces (same as in **B**, left). Scale bars for inset are 1% ΔF/F and 0.2 s. Both Y522A^*^ and Y522F^*^ show statistically significant voltage-dependent shifts of activity to higher voltages (Table 1), *n* ≥ 6 (**C**, left) Representative TMRM fluorescence trace during a step from an hp of −80 to +200 mV for WT^*^ and Y522F^*^. Colors as in **(A)**. Traces are normalized to the maximal fluorescence change. The voltage trace reports the actual voltage recorded during acquisition. Y522F^*^ causes a small deceleration of the VSD activation relative to WT^*^. (**C**, right) Normalized TMRM FV. Data fit to single Boltzmann equation and normalized to the fit. Y522F^*^ shifted the voltage dependence of the VSD motions by over 20 mV, *n* ≥ 10, WT^*^ *V*_1/2_ = 55.1 ± 0.4, slope = 23.9 ± 0.4; Y522F^*^ *V*_1/2_ = 77.8 ± 0.6, slope = 26.8 ± 0.5. All error bars are ± s.e.m. Some errors bars are smaller than the size of the symbols.

The contribution of the C2 domain to Ci-VSP activity was first discovered with the crystal structures, where Y522 on the third inactivated CBL (iCBL3) of the C2 was found pointing into the active site (Matsuda et al., 2011; Liu et al., 2012). The Y522 was mutated to an alanine (Ala) and shown to influence activity by slowing the rate of PI(3,4)P_2_ dephosphorylation (Liu et al., 2012). We applied our wider voltage range PH domain assay to Y522A^*^ (G214C Y522A labeled with TMRM) to further probe the role of this key residue. We observe both 5- and 3-phosphatase activities for Y522A^*^, though the equilibrium of net reactions are altered (Figure 2). When GFP-TAPP-PH was co-expressed with Y522A^*^, a representative 70 mV step shows a mixture of increasing and decreasing fluorescence suggesting both 5- and 3-phosphatase activities (Figure 2A, left, magenta) as compared to WT^*^ that shows a predominant decrease (Figure 2A, left, black). Plotting the TAPP FV shows that at higher voltages, the fluorescence decrease of Y522A^*^ became even more pronounced, indicating that Y522A^*^ still enters a dominant 3-phosphatase activity state (Figure 2A, right, magenta). As seen before (Liu et al., 2012), Y522A^*^ produced a larger amplitude increase in fluorescence at 20 mV indicating higher PI(3,4)P_2_ concentrations on the membrane compared to WT^*^ (statistically significant, Student's *t*-test, *p* ≤ 0.05). The broader voltage range used here shows that the increase is maintained even at higher voltages suggesting that the slower PI(3,4)P_2_ dephosphorylation is consistent across voltages. When fitting the FVs to a double Boltzmann, the *V*_1/2_ values were not statistically different between WT^*^ and Y522A^*^ indicating the voltage dependence of the reactions was not altered (Table 1) even when the relative rates between the reactions were different. The mechanism behind Y522 is still unknown so we repeated our experiments with a phenylalanine mutation to retain the size of the ring, but remove the hydrogen bonding capability. Interestingly, the Y522F^*^ (G214C Y522F labeled with TMRM) activity does not show the same larger amplitude signal above WT^*^ levels suggesting the rates are the same, however Y522F^*^ clearly shifted the voltage dependence by almost 20 mV (Figure 2A, right, green, Table 1) suggesting that hydrogen bonding may be a factor for Y522 influence on the voltage dependence of catalysis independent of the size of the side chain.

When testing the PI(4,5)P_2_ pathway using GFP-PLC-PH, we observed a different trend with Y522A^*^. The amplitudes of Y522A^*^ were not significantly different in the fluorescence decrease nor in the increase consistent with previous studies (Liu et al., 2012). Fitting the PLC FV Down revealed no significant difference between the voltage dependence of Y522A^*^ compared to WT^*^ (Figure 2B, left). The PLC FV Up did reveal a small difference between Y522A^*^ and WT^*^ with the *V*_1/2_ shifted by 17 mV when the data were fit with a single Boltzmann (Figure 2B, right, Table 1). Y522F^*^, on the other hand, shifted both the PLC FV Down and Up to higher voltages by 20 mV (Figure 2B, Table 1) suggesting both 3- and 5-phosphatase activities were changed. Overall, Y522A^*^ changed the activity of VSP in two ways, by changing the reaction rates of the PI(3,4)P_2_ pathway while shifting the 3-phosphatase reaction of the PI(4,5)P_2_ pathway to higher energy suggesting that the Y522 position plays a role in the substrate selectivity of VSP. Y522F^*^ had a stronger influence on activity even though it is the more conservative mutation, shifting the voltage dependence of both reaction pathways suggesting that the hydrogen bonding capability of the Y522 position strongly influences the voltage dependence of activity.

We also tested how these mutations altered another aspect of VSP function, the motions of the VSD. The G214C labeling site has been well-established to monitor the motions of the VSP S4 helix by labeling with TMRM and observing the changes in TMRM fluorescence using VCF (Kohout et al., 2008; Villalba-Galea et al., 2008). Interestingly, while Y522A^*^ accelerated the VSD activation and deactivation kinetics (Liu et al., 2012), Y522F^*^ had only a minor effect on the activation kinetics (Figure 2C, left). Y522F^*^ significantly shifted the voltage dependence of those motions (Figure 2C, right) suggesting that maintaining the size of the side chain and only changing the hydrogen bonding capability influences the voltage dependence of VSP function more than removing both the size and the hydrogen bonding. The PH domain assay combined with the TMRM VCF data strongly suggest other factors may be involved in the mechanism of action for Y522.

### Deleting the C2 domain eliminates activity

**Figure 3:**
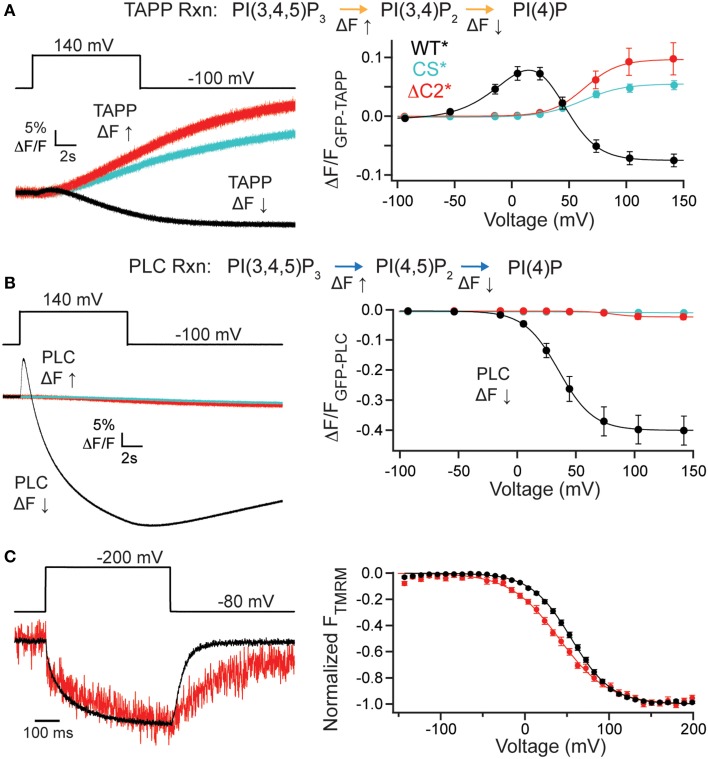
**C2 domain required for VSP activity**. (**A**, left) Representative GFP-TAPP-PH trace during a single voltage step from an hp of −100 mV to +140 mV for WT^*^ (black), ΔC2^*^ (red), and CS^*^ (cyan). ΔC2^*^ shows minimal activity above endogenous levels (CS^*^). TAPP-PH reaction listed on top. (**A**, right) ΔF/F TAPP FV. ΔC2^*^ activity not significantly different from CS^*^ indicating only endogenous activity even at the highest voltage recorded, *n* ≥ 9. Data fit with a single Boltzmann. (**B**, left) Representative GFP-PLC-PH co-expressed with WT^*^, ΔC2^*^, and CS^*^ during a +140 mV step. WT^*^ shows the characteristic increase and decrease in fluorescence indicating both PI(4,5)P_2_ production and depletion while ΔC2^*^ and CS^*^ show almost no change in fluorescence. PLC-PH reaction listed on top. (**B**, right) ΔF/F PLC FV Down (net 5-phosphatase reaction). ΔC2^*^ shows no activity (either 3- or 5-phosphatase) beyond that of endogenous VSPs in the oocytes. Colors as in **(A)**, *n* ≥ 9. (**C**, left) Representative TMRM fluorescence trace during a step from a hp = −80 to +200 mV for WT^*^ and ΔC2^*^. Traces are normalized to the maximal fluorescence change. The voltage trace reports the actual voltage recorded during acquisition. ΔC2^*^ has similar VSD activation kinetics to WT^*^ but slower deactivation kinetics. (**C**, right) Normalized TMRM FV. Data fit to single Boltzmann equations and normalized to the fit. The ΔC2^*^ voltage dependence is shifted to lower voltages. WT^*^ *V*_1/2_ = 55.1 ± 0.4, slope = 23.9 ± 0.4; ΔC2^*^ *V*_1/2_ = 41 ± 1, slope = 29 ± 1. Error bars are ± s.e.m., *n* ≥ 11. Some errors bars are smaller than the size of the symbols.

It is possible that the C2 domain may modulate activity while not being necessary for activity. To address this, we deleted the entire C2 domain (ΔC2^*^, G214C deleted C2 labeled with TMRM), truncating the protein at amino acid 432 and tested both PI(3,4)P_2_ and PI(4,5)P_2_ reaction pathways. Starting with the PI(3,4)P_2_ pathway, we co-expressed GFP-TAPP-PH with ΔC2^*^ and observed a robust rise in fluorescence at +140 mV (Figure 3A, left, red). That rise was similar to the fluorescence increase of a catalytically dead Ci-VSP, C363S (CS^*^, G214C C363S labeled with TMRM) used as a control (Figure 3A, left, cyan). This is because *X. laevis* oocytes express two endogenous VSPs, Xl-VSP 1 and 2 (Ratzan et al., 2011; Liu et al., 2012). Both ΔC2^*^ and CS^*^ were distinct from WT^*^ where the fluorescence predominantly decreases at that voltage (Figure 3A, left, black). Over the full range of voltages, ΔC2^*^ induced only fluorescence increases similar to those seen for CS^*^ with the same voltage dependence (Figure 3A, right, Table 1). Interestingly, some ΔC2^*^ cells gave slightly higher magnitude changes in fluorescence which might imply a small degree of activity above control. However, those differences were never statistically different from CS^*^ (Figure 3A, right), indicating ΔC2^*^ is not functional.

We observed similar results for the PI(4,5)P_2_ pathway tested with GFP-PLC-PH. ΔC2^*^ caused a small fluorescence decrease during the +140 mV step, similar to that of CS^*^ and in sharp contrast to the dramatic decrease of WT^*^ (Figure 3B, left). No fluorescence increase was ever observed for ΔC2^*^, also similar to CS^*^ control (Figure 3B, left). The PLC FV Down also shows a slightly larger magnitude change in the fluorescence for ΔC2^*^ over the larger voltages, however, they are not statistically different from CS^*^ (Figure 3B, right), indicating that ΔC2^*^ is no longer active against any of its substrates. The signals for both CS^*^ and ΔC2^*^ with GFP-PLC-PH were very small, often < 1% ΔF/F, making fitting that data unreasonable. As with the other mutants, we confirmed the expression of ΔC2^*^ using TMRM and VCF. ΔC2^*^ still expressed, indicating the lack of activity is not due to lack of protein and VCF results showed similar VSD activation kinetics, while the VSD deactivation kinetics were slower (Figure 3C, left). The voltage dependence of ΔC2^*^ VSD motions were slightly shifted to *V*_1/2_ = 41 ± 1 as compared to WT^*^, *V*_1/2_ = 55.1 ± 0.3. This shift can be partly attributed to a change in the slope (ΔC2^*^ slope = 29 ± 1; WT^*^ slope = 23.9 ± 0.4) suggesting the voltage dependence is not as pronounced (Figure 3C, right). Though less energy is needed to move the VSD, it takes longer for the VSD to recover to a resting state when the C2 domain has been deleted. These changes in kinetics and voltage dependence show that the C2 domain influences the overall function of VSP, both the motions of the VSD as well as catalysis.

### C2 domain positive charge cluster influences VSP activity

**Figure 4:**
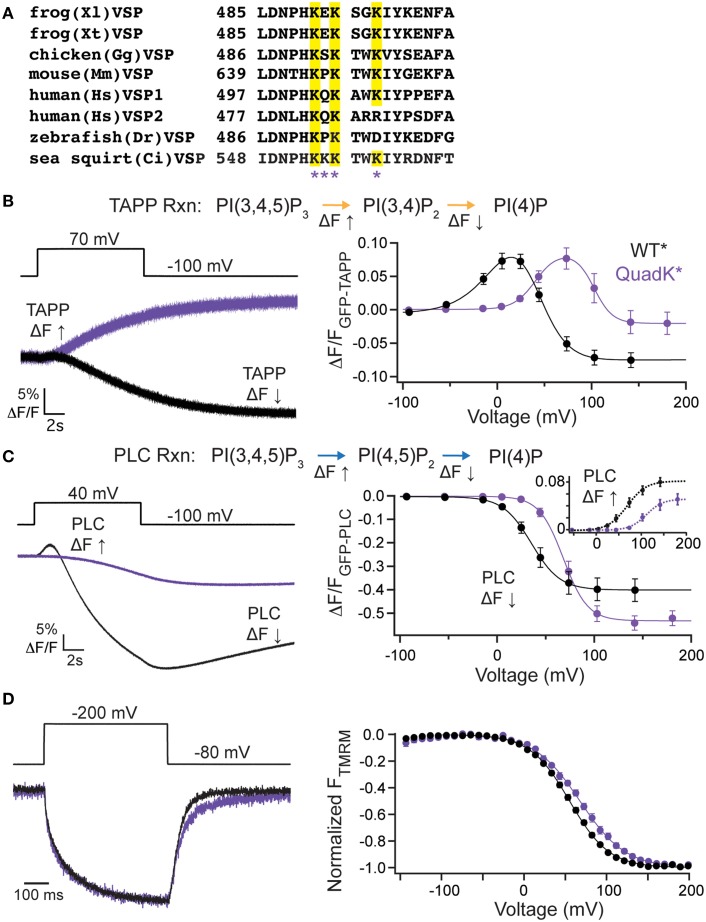
**C2 domain positive charge cluster significantly shifts voltage-dependent activity**. (**A**, left) Alignment of the fourth inactivated CBL from several species of VSP. Quadruple Lys mutant, quadK^*^, positions highlighted by asterisks. Conserved Lys shown in yellow. (**B**, left) Representative GFP-TAPP-PH trace during a step from an hp of −100 mV to +70 mV for WT^*^ (black) and quadK^*^ (purple). QuadK^*^ shows significant levels of PI(3,4)P_2_ production while WT^*^ shows predominantly PI(3,4)P_2_ depletion during a 70 mV step. TAPP-PH reaction listed on top. (**B**, right) ΔF/F TAPP FV. QuadK^*^ significantly shifts the PI(3,4)P_2_ production and depletions to higher voltages (>40 mV, Table 1), *n* ≥ 6. Data fit with a double Boltzmann. (**C**, left) Representative GFP-PLC-PH trace during a step from an hp of −100 mV to +40 mV for WT^*^ and quadK, colors as in **(B)**. QuadK^*^ again shows a different level of PI(4,5)P_2_ production as compared to wild type. PLC-PH reaction listed on top. (**C**, right) ΔF/F PLC FV with the fluorescence decrease (Down, net 5-phosphatase reaction) in solid lines and the fluorescence increase shown in inset with dashed lines (Up, net 3-phosphatase reaction). QuadK^*^ significantly shifted the PI(4,5)P_2_ production and depletion to higher voltages (>30 mV, Table 1), *n* ≥ 7. Data fit with a Boltzman. (**D**, left) Representative TMRM fluorescence trace during a step from an hp of −80 to +200 mV for WT^*^ and quadK^*^. Colors as in **(B)**. Traces are normalized to the maximal fluorescence change. The voltage trace reports the actual voltage recorded during acquisition. QuadK^*^ does not significantly influence the VSD activation kinetics while slowing the deactivation kinetics modestly. (**D**, right) Normalized TMRM FV. A small (12 mV) shift in the voltage dependence of VSD motions with quadK^*^. Data fit to single Boltzmann equations and normalized to the fit, *n* ≥ 10, WT^*^ *V*_1/2_ = 55.1 ± 0.4, slope = 23.9 ± 0.4; quadK^*^ *V*_1/2_ = 67.6 ± 0.7, slope = 27.4 ± 0.6. All error bars are ± s.e.m. Some errors bars are smaller than the size of the symbols.

While the VSP C2 contribution to catalysis is unique, the long established role of C2 domains is to bind lipids (Nalefski and Falke, 1996; Lemmon, 2008) even when calcium is not involved as in the case of protein kinase C ε (PKCε) and PTEN C2 domains (Corbalán-García et al., 2003; Sánchez-Bautista et al., 2007). Simulation data suggests that a cluster of positive charges on the VSP C2 could be contributing to binding PI(4,5)P_2_ lipids (Kalli et al., 2014). Four of these positive charges are found in the fourth CBL (Figure 4A). To test whether these positive charges play a role in VSP function, we mutated all four lysines (Lys), K553, K554, K555, and K558, to glutamine (Gln) (quadK^*^, G214C K553-5Q K558Q labeled with TMRM), neutralizing all four positive charges simultaneously. When tested with GFP-TAPP-PH, we observed a significant shift in the voltage-dependent activity; at +70 mV, quadK^*^ still displayed a strong fluorescence increase in contrast to the clear fluorescence decrease displayed by WT^*^ (Figure 4B, left). QuadK^*^ was still capable of inducing a fluorescence decrease in response to a voltage pulse, but required >100 mV before it dominated the net reactions (Figure 4B, right). Overall, the activity of quadK^*^ required higher energy with both TAPP FV Up and Down shifted to higher voltages by ≥40 mV (Table 1). The 5-phosphatase reaction had similar amplitudes between WT^*^ and quadK^*^, but the 3-phosphatase reaction showed a smaller amplitude change suggesting that some reaction rates may be altered in quadK^*^. We found similar results when testing the PI(4,5)P_2_ pathway with GFP-PLC-PH. At a representative 40 mV, quadK^*^ shows a significantly smaller fluorescence decrease and no fluorescence increase compared to WT^*^ (Figure 4C, left). However, at larger voltages, both reactions are clearly visible, though shifted by ≥30 mV (Figure 4C, right, Table 1). The overall amplitude of the GFP-PLC-PH fluorescence was larger for quadK^*^ when monitoring the fluorescence decrease and smaller when monitoring the fluorescence increase (Figure 4C, right) supporting the hypothesis that quadK^*^ has also shifted the relative reaction rates of VSP. Using VCF, we observed that the quadK^*^ had only minor effects on the deactivation kinetics (Figure 4D, left), while the voltage dependence of the VSD motions were shifted by >10 mV to higher voltages (Figure 4D, right). These lysines are not likely to interact with the bound substrate as seen by the crystal structure (Liu et al., 2012). Instead they may be modulating the voltage dependence of voltage sensor movements and activity either by allosteric effects or by changing the face of the protein that interacts with the membrane.

**Figure 5:**
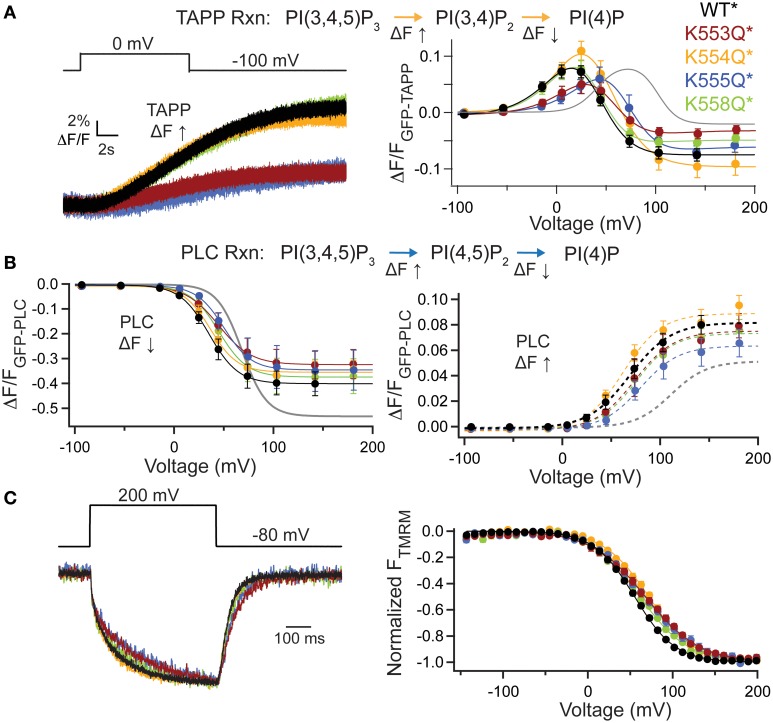
**Individual mutations in the C2 positive charge cluster shift some, not all activity**. (**A**, left) Representative GFP-TAPP-PH trace during a step from an hp of −100 mV to 0 mV for WT^*^ (black), K553Q^*^ (rust), K554Q^*^ (orange), K555Q^*^ (blue), and K558Q^*^ (green). Both K553Q^*^ and K555Q^*^ show a smaller change in fluorescence compared to WT^*^, K554Q^*^, and K558Q^*^, indicating less PI(3,4)P_2_ production at 0 mV. TAPP-PH reaction listed on top. (**A**, right) ΔF/F TAPP FV. Both K553Q^*^ and K555Q^*^ show significant shifts in the voltage dependence of activity (10–30 mV) and their sum recapitulates the net 5-phosphtase shift from quadK^*^, but not the net 3-phosphatase shift (gray line shows quadK^*^), *n* ≥ 6. Data fit with a double Boltzmann. (**B**, left) ΔF/F PLC FV Down (net 5-phosphatase activity) from GFP-PLC-PH co-expressed with individual Lys to Gln mutations, fit with a single Boltzmann. Any shift in the voltage dependence is not significant (Table 1). Colors as in **(A)**, *n* ≥ 6. PLC-PH reaction listed on top. (**B**, right) ΔF/F PLC FV Up (net 3-phosphatase activity) from the same GFP-PLC-PH data as in (**B**, left), fit with a single Boltzmann. Again, none of the *V*_1/2_ shifts are significant (Table 1), *n* ≥ 6. (**C**, left) Representative TMRM fluorescence trace during a step from an hp of −80 to +200 mV for WT^*^, K553Q^*^, K554Q^*^, K555Q^*^, and K558Q^*^. Colors are as in **(A)**. Traces are normalized to the maximal fluorescence change. The voltage trace reports the actual voltage recorded during acquisition. K553Q^*^ and K555Q^*^ slightly slow down the activation and deactivation kinetics of the VSD motions. (**C**, right) Normalized TMRM FV. All Lys to Gln mutations shifted the VSD motions by 6–17 mV to higher voltages. Data fit to single Boltzmann equations and normalized to the fit, *n* ≥ 9. WT^*^ *V*_1/2_ = 55.1 ± 0.4, slope = 23.9 ± 0.4; K553Q^*^ *V*_1/2_ = 68 ± 1, slope = 32 ± 1; K554Q^*^ *V*_1/2_ = 71.8 ± 0.8, slope = 26.6 ± 0.7; K555Q^*^ *V*_1/2_ = 67.3 ± 0.9, slope = 30.8 ± 0.9; K558Q^*^ *V*_1/2_ = 61.3 ± 0.9, slope = 28.7 ± 0.9. All error bars are ± s.e.m.

To further dissect the contributions of these lysines, we individually mutated them to Gln. The alignment shows that K553 and K555 are the most well-conserved across several VSP species, K558 is somewhat conserved while K554 is not conserved at all (Figure 4A). We co-expressed the individual Lys to Gln mutations with either GFP-TAPP-PH (Figure 5A) or GFP-PLC-PH (Figure 5B) to determine their activities relative to WT^*^ and quadK^*^. For GFP-TAPP-PH, the individual mutants K553Q^*^ and K555Q^*^ show a different amplitude change in the fluorescence for a representative 0 mV step, compared to WT^*^, K554Q^*^, and K558Q^*^ (Figure 5A, left). This segregation continues in the full FVs, where K553Q^*^ and K555Q^*^ each show a shift in the fluorescence to higher voltages for the TAPP FV Up and Down while K554Q^*^ and K558Q^*^ are not statistically different from WT^*^ in either direction (Figure 5A, right, Table 1). The sum of the FV shift for K553Q^*^ and K555Q^*^ equals the shift for quadK^*^ when looking at the TAPP FV Up reaction but not for the TAPP FV Down reaction (Figure 5A, right, Table 1). For co-expression with GFP-PLC-PH, we observe a different trend where the minor changes observed in the PLC FV Down and Up for the individual mutations were not statistically different compared to WT^*^ (Figure 5B, Table 1). The sum of the amplitude from each individual Lys to Gln mutant also did not recapitulate the amplitude changes seen with quadK^*^ for the PI(4,5)P_2_ pathway. Both the TAPP and PLC data indicate that the quadK^*^ shift may be more than just the sum of the individual Lys mutants and implies other factors may contribute. The VSD motions measured by VCF were only slightly altered (Figure 5C, left) while all the TMRM FVs were shifted to higher voltages by 6–17 mV (Figure 5C, right). The TMRM data for the individual Lys to Gln mutations again does not recapitulate the quadK^*^ TMRM data since K553Q^*^, K554Q^*^, and K555Q^*^ all shift the TMRM FV by almost the same amount as quadK^*^ (*V*_1/2_'s are not statistically different between quadK^*^ and K553-5Q^*^). The TMRM data combined with the PH domain data indicates that the shift seen from quadK^*^ is not the simple sum of the individual Lys positions and other factors are influencing VSP through those positive charges.

### Lipid contribution to C2 domain function

**Figure 6:**
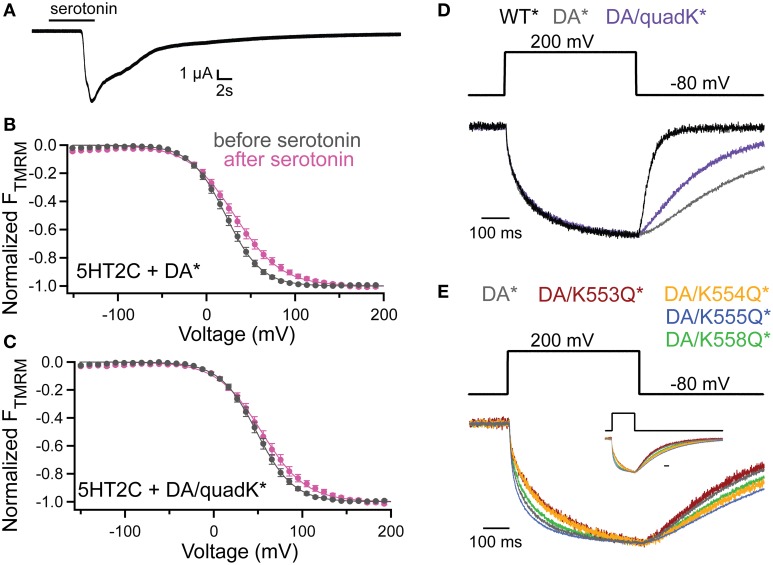
**PI(4,5)P_2_ depletion does not alter voltage dependence of VSD motions. (A)** Current trace showing the activation of the endogenous Ca^2+^-activated chloride channel following addition of serotonin in oocytes expressing the 5HT2C receptor and Ci-VSP. 5HT2C receptor activation of the oocyte PLC pathway results in the conversion of PI(4,5)P_2_ into IP_3_ and DAG. The IP_3_ causes an increase in intracellular Ca^2+^ leading to the activation of the chloride channel. hp = −80 mV. **(B,C)** TMRM FVs before (black) and 10 min after (magenta) application of 10 μM serotonin. Activation of PLC through the 5HT2C receptor leads to depletion of PI(4,5)P_2_ in the membrane and results in a shift in the DA^*^ **(B)** and DA/quadK^*^ **(C)** TMRM FVs after addition of serotonin. In both cases, the VSD motions are sensitive to the PI(4,5)P_2_ in the membrane, indicating that the DA/quadK^*^ may not depend on PI(4,5)P_2_ in the membrane to cause the activity shifts seen in Figure 4. DA^*^ (no serotonin) *V*_1/2_ = 21.8 ± 0.3, slope = 19.9 ± 0.2; DA^*^ (serotonin) *V*_1/2_ = 31.7 ± 0.6, slope = 26.2 ± 0.5; DA/quadK^*^ (no serotonin) *V*_1/2_ = 48.3 ± 0.2, slope = 20.7 ± 0.2; DA/quadK^*^ (serotonin) *V*_1/2_ = 54.0 ± 0.6, slope = 25.0 ± 0.5. All error bars are ± s.e.m. **(D)** Representative TMRM fluorescence traces during a step from an hp of −80 to +200 mV for WT^*^ (black), DA^*^(gray), and DA/quadK^*^ (purple). Traces are normalized to the maximal fluorescence change. DA^*^ significantly slows down the repolarization kinetics while DA/quadK^*^ kinetics were faster. **(E)** Representative TMRM fluorescence traces during a step from an hp of −80 to +200 mV for DA^*^ (black), DA/K553Q^*^ (rust), DA/K554Q^*^ (orange), DA/K555Q^*^ (blue), and DA/K558Q^*^ (green). Traces are normalized to the maximal fluorescence change. The voltage trace reports the actual voltage recorded during acquisition. Inset shows full acquisition with the fluorescence returning to baseline. Scale bar for inset is 100 ms. All Lys to Gln mutations in the D331A background maintained the slow or slower kinetics from DA^*^ alone. The individual mutations do not sum to equal the effect observed for DA/quadK^*^ in **(D)**.

To probe whether the activity shifts seen with the Lys mutants stem from an interaction with the membrane through PIPs, we co-expressed quadK^*^ with the serotonin 2C receptor (5HT2C). The 5HT2C G protein coupled receptor activates endogenous phospholipase C (PLC), leading to the cleavage of the PI(4,5)P_2_ phosphodiester bond creating diacylglycerol (DAG) and inositol-(1,4,5)-trisphosphate (IP_3_), effectively removing PI(4,5)P_2_ from the membrane (Julius et al., 1988). Previous results have shown that Ci-VSP VSD motions are dependent on PI(4,5)P_2_ in the membrane using the 5HT2C receptor (Kohout et al., 2010). Here we used the same background mutation, D331A, as in the previous study (Kohout et al., 2010) because it inactivates VSP catalysis simplifying the interpretation of results by removing VSP-induced PIP depletion. After Ci-VSP co-expression with 5HT2C, the TMRM FV is recorded before the addition of the serotonin, under native PI(4,5)P_2_ concentration. Then, in the same cell, the addition of 10 μM serotonin depletes the PI(4,5)P_2_ and after 10 min, we re-measure the FV. We confirmed the activation of the PLC pathway by recording the calcium-activated chloride channels that are natively expressed in oocytes (Figure 6A). These channels open as a result of the IP_3_-induced calcium release from the internal stores of the cell. We repeated the previously published results using DA^*^ (G214C D331A labeled with TMRM) and found a robust shift in the TMRM FV after addition of serotonin and PI(4,5)P_2_ depletion (Figure 6B). We conducted the same experiment with DA/quadK^*^ (G214C D331A quadK labeled with TMRM) and found that the FV still shifts after depletion of PI(45)P_2_ in the membrane (Figure 6C) indicating that those positive charges are not contributing to the PI(4,5)P_2_-induced voltage-dependent VSD rearrangements. If these C2 Lys residues interact with the membrane, it maybe be through a combination of negatively charged lipids instead of exclusive interactions with PI(4,5)P_2_.

Interestingly, the DA/quadK^*^ disrupted the characteristic slower repolarization kinetics for DA^*^, making them faster (Figure 6D). The individual Lys to Gln, on the other hand, gave the same slow kinetics or even slower kinetics than DA^*^ alone (Figure 6E). The influence of the DA/quadK^*^ on VSD kinetics, but not of the individual mutations, further supports the idea that the effects from quadK^*^ are more than the sum of the individual mutations and other factors are influencing the motions. Further experiments are needed to fully explore potential membrane interactions with these residues.

## Discussion

We have probed the contributions of the VSP C2 domain by testing catalysis, motions of the VSD, and the influence of the membrane. We have found that the VSP C2 has a strong influence on activity through both direct and indirect contributions to the active site. The indirect influence may be allosteric or may be through interactions with the membrane. In the past, much of the field has concentrated on understanding how the VSD or how the linker works in VSP. Through our findings, the C2 domain has emerged as a critical factor for understanding both catalysis and membrane interactions of VSP.

When testing for activity, we used the PH domain system for detecting membrane levels of PIPs. The assay allows us to observe changing PIP concentrations by monitoring the movement of the PH domain to and from the membrane. The changing fluorescence can be attributed to the appropriate voltage-dependent Ci-VSP activity, either 3- or the 5-phosphatase. This interpretation does not preclude other complicating factors including the possibility that the changing PIP concentrations themselves are influencing the reactions or the contributions of the native kinases and phosphatases within the cell. By testing a broad range of voltages we were able to observe the fluorescence increases and decreases of each reporter, determine the voltage dependence of those changes and attribute them to either the 5- or 3-phosphatase activity of Ci-VSP. By doing so, we observed the disputed 3-phosphate dephosphorylation of PI(3,4,5)P_3_ in a voltage-dependent manner. Our results provide evidence for Ci-VSP 3-phosphatase activity against two substrates, PI(3,4)P_2_ in agreement with previous reports (Kurokawa et al., 2012; Liu et al., 2012) and PI(3,4,5)P_3_, which has not been clearly shown before. Thus, wild type Ci-VSP is capable of dephosphorylating three different substrates, PI(3,4,5)P_3_, PI(4,5)P_2_, and PI(3,4)P_2_ via four different reactions (Figure 1B). Studies in HEK293 cells have not observed the 3-phosphatase reaction which perhaps indicates a different environment between HEK293 cells and *X. laevis* oocytes (Halaszovich et al., 2009). It is important to note that other studies using GFP-PLC-PH in oocytes have not observed the fluorescence rise indicating 3-phosphatase activity against PI(3,4,5)P_3_ (Kurokawa et al., 2012). This may be due to a functional limitation of the 3-phosphatase activity. We only observed it at higher voltages (≥40 mV) and it may easily get masked by the strong fluorescence decrease from the 5-phosphatase activity.

All the crystal structures solved to date place the Y522 residue in the active site (Matsuda et al., 2011; Liu et al., 2012). By mutating Y522 to either an Ala or a Phe, we were able to probe what factors are involved in the Y522 contribution to catalysis. We found that both size and hydrogen bonding may play a role. Unexpectedly, Y522F^*^ shifted the voltage dependence of activity and VSD motions more than Y522A^*^ indicating that by removing the OH alone, higher voltages are needed to achieve the same level of function, but by removing both the phenyl ring and the OH group, the voltages were closer to wild type. The crystal structure active site shows a histidine residue, H332, within 4 Å of Y522. It is possible that by removing the hydrogen bonding, Y522F is more likely to form a π-cation or a π−π interaction between the Y522F and H332. This interaction would need to be broken for catalysis to progress, thus leading to a larger shift where higher voltage is necessary to achieve the same level of function, either catalysis or VSD motions. The native Tyr would have electron density pulled away from the aromatic ring toward the active site and the Ala would not contribute to any interaction and thus would not require the extra energy of activation. Further experiments will be needed to probe this hypothesis.

Previous results using a 20 mV voltage pulse showed that Y522A decreased the reaction rate for the 3-phosphate dephosphorylation of PI(3,4)P_2_ resulting in increased accumulation of PI(3,4)P_2_ on the membrane over wild type (Liu et al., 2012). We see that same effect over a broad range of voltages, from 20 to 70 mV, for Y522A^*^ but not for Y522F^*^ suggesting that the size of the amino acid at position 522 influences 3-phosphatase activity rate while the hydrogen bonding alone does not. At ≥70 mV, a net fluorescence decrease predominates suggesting 3-phosphatase activity for both mutations indicating that even with slower reaction rates, the 3-phosphatase reaction is still capable of dominating. Y522 is conserved across all VSPs with C2 domains and our results suggest the 522 position functions as a tuner for the reaction rates instead of an absolute regulator.

While it is clear that Y522 has defined a new role for C2 domains in catalysis, whether the C2 domain was required for activity was still in question. We deleted the entire C2 domain starting at amino acid 432 and found that it does not show any statistically significant activity above the endogenous levels of the Xl-VSP1 and 2. Interestingly, there were a few cells for both GFP-TAPP-PH and GFP-PLC-PH that showed greater than endogenous levels of activity suggesting a very low level of activity. However, with a larger sample size, the resulting ΔF/Fs were no longer significant. There may be very low levels of activity that are hard to distinguish from endogenous. ΔC2^*^ also influenced the voltage-dependent motions of the VSD, slowing down the repolarization kinetics while shifting the voltage dependence to lower voltages. While the majority of VSPs have C2 domains, a recent report discovered that the salamander VSP (*Hynobius nebulosus*, Hn-VSP) has a truncated C2 stopping at CBL2 (Mutua et al., 2014). They found no activity for Hn-VSP using GIRK channels and when they truncated Ci-VSP at the same position, they also inactivated Ci-VSP. Our results combined with those from Mutua et al. strongly suggest that the C2 domain plays an active and necessary role within VSP function.

An important aspect of C2 domains is their ability to bind membranes. Modeling studies have suggested that the C2 domain from VSP is no different and identified a cluster of Lys residues that may be involved in interacting with the negatively charged lipid PI(4,5)P_2_ (Kalli et al., 2014). We mutated four of those Lys residues simultaneously to determine whether any functions of VSP, either activity or the motions of the VSD, were altered as a result. We found a significant shift (30–40 mV) in the voltage dependence of activity for both 5- and 3-phosphatase reactions as monitored by both GFP-TAPP-PH and GFP-PLC-PH. The crystal structures illustrate that the shift cannot be a result of a direct interaction with the bound substrate, thus the effect must be indirect (Matsuda et al., 2011; Liu et al., 2012). The VSD motions were also altered, with a shift to higher energy in the voltage dependence of the motions. We mutated the four Lys residues individually to further dissect the effect and found while some shifted the activity to higher energy, others did not. The shifts were not consistent between the GFP-TAPP-PH and GFP-PLC-PH experiments and of those that did shift, they did not fully recapitulate the large shift from the quadK^*^. The VSD motions also did not recapitulate the quadK^*^ shift where some individual mutants shifted as far as the quadK^*^. Together, these results indicate that the individual Lys mutants are behaving differently than when they are all mutated together suggesting a synergistic effect between the four. This synergistic effect may be allosteric in nature or due to another player, the membrane.

To address whether the membrane may be interacting with this positive cluster, we used the serotonin receptor to deplete membrane PI(4,5)P_2_ concentrations, the lipid that has been suggested to interact with this region (Kalli et al., 2014). Previous results have shown that the VSD motions are sensitive to PI(4,5)P_2_ concentrations, so we used the same VCF assay to monitor the voltage-dependent motions of the VSD before and after PI(4,5)P_2_ depletion. DA/quadK^*^ still shifted the voltage dependence of the VSD motions even after PI(4,5)P_2_ depletion suggesting those Lys residues are not involved in the shift. These results do not negate the C2 interaction with the membrane however. One possibility is an interaction with negatively-charged phosphatidylserine, which has been shown to interact with the PTEN C2 domain (Shenoy et al., 2012a,b; Nanda et al., 2014). Other negatively charged lipids such as other VSP substrates, PI(3,4,5)P_3_ and PI(3,4)P_2_, may also be involved. Other lipids are not tested here since the 5HT2C receptor activates PLC specifically targeting PI(4,5)P_2_ and not other lipids. Further experiments are needed to determine the membrane-binding contribution of the VSP C2 domain.

While it is clear from our studies that Y522 and the polylysine cluster play a significant role in VSP function, the mutations tested here do not recapitulate the loss of function seen when the entire C2 domain is deleted. Other aspects of the VSP C2 may contribute to VSP catalysis and lipid-binding while other roles for the C2 may also exist that have not been tested, such as lipid shuttling to the active site or protein–protein interactions. Other C2 domains have been shown to mediate protein–protein interactions, such as the synaptic RIM (Rab3-interacting molecule). This protein has two C2s that fold in the canonical β-sandwich, however disease mutations are found on the bottom face of the protein, away from the CBLs and structural studies suggest that the protein-binding region is Ca^2+^-independent and mediated by the bottom face (Dai et al., 2005). The PKCε C2 has also been shown to function as a protein interaction domain (Johnson et al., 1996; Schechtman et al., 2004). Thus, further experiments are needed to fully understand how the C2 domain is contributing to VSP function.

**Figure 7:**
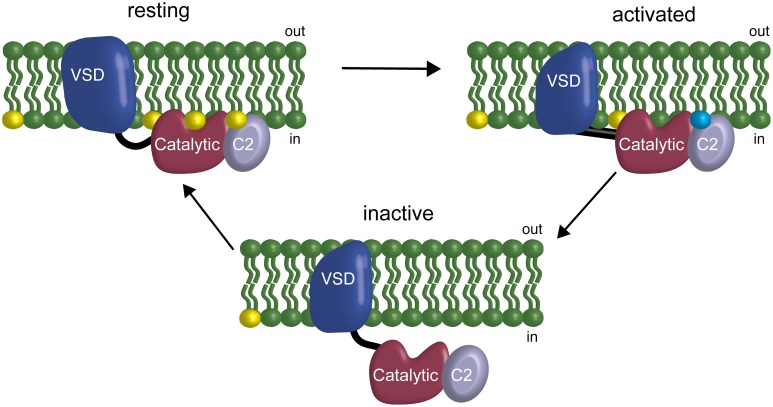
**Model of the C2 domain interaction with the membrane and influence in a feedback mechanism**. At resting membrane potentials, the phosphatase domain is on the membrane but inactive. At activating membrane potentials, the VSD changes the conformation of the linker allowing activation of the active site and starts the depletion of the PIPs on the membrane. If the VSD does not deactivate, the subsequent depletion of PIPs leads the phosphatase domain to unbind from the membrane, effectively turning off catalysis without changing the activation state of the VSD. This feedback is driven by the phosphatase domain interacting with the membrane via PIPs at the linker (Kohout et al., 2010), the catalytic domain and potentially the C2 domain. Yellow indicates PI(4,5)P_2_ in the membrane. Blue indicates unknown negatively charged lipid in the membrane that could be one of the other substrates for VSP: PI(3,4,5)P_3_ or PI(3,4)P_2_.

Previous results have suggested a VSP feedback mechanism wherein VSP is modulated by the very lipids it uses as substrates, specifically suggesting that the linker between the VSD and the catalytic domain mediates the feedback (Kohout et al., 2010). Our results here indicate that the C2 domain is also involved in modulation of activity through both direct and indirect interactions. The direct interaction, Y522, modulates rates and voltage dependence of catalysis. The indirect interactions, the cluster of positive charges on the fourth CBL, also shift the voltage dependence of catalysis. This shift may be due to interactions with negatively charged lipids in the membrane and thus be part of the feedback mechanism though perhaps not through PI(4,5)P_2_ (Figure 7). This model of VSP function suggests a coincident detection mechanism in play as well as a feedback mechanism where more than one lipid is detected between the linker and the C2 to modulate the binding of the catalytic domain to the membrane. Together, our results show that the C2 domain actively contributes to VSP function because it modulates the rate and voltage dependence of catalysis either through direct or indirect mechanisms.

## Author contributions

PMC, KDZ and SCK conceived of the study, performed the experiments, analyzed data, and wrote the paper.

### Conflict of interest statement

The authors declare that the research was conducted in the absence of any commercial or financial relationships that could be construed as a potential conflict of interest.
